# Joint European Society of Paediatric Radiology (ESPR) and International Society for Forensic Radiology and Imaging (ISFRI) guidelines: paediatric postmortem computed tomography imaging protocol

**DOI:** 10.1007/s00247-018-04340-x

**Published:** 2019-02-28

**Authors:** Susan C. Shelmerdine, Chandra Y. Gerrard, Padma Rao, Matthew Lynch, Jeroen Kroll, Dan Martin, Elka Miller, Laura Filograna, Rosa Maria Martinez, Odey Ukpo, Barry Daly, Hideki Hyodoh, Karl Johnson, Andrew Watt, Ajay Taranath, Scott Brown, David Perry, Lene Warner Thorup Boel, Aleksandra Borowska-Solonynko, Rick van Rijn, Willemijn Klein, Elspeth Whitby, Owen J. Arthurs

**Affiliations:** 1 Department of Clinical Radiology, Great Ormond Street Hospital for Children, London, UK; 2 UCL Great Ormond Street Institute of Child Health, Great Ormond Street Hospital for Children, London, UK; 3 Department of Radiology, University of New Mexico, Albuquerque, NM USA; 4 Department of Medical Imaging, Victorian Institute of Forensic Medicine & Royal Children’s Hospital, Melbourne, Australia; 5 Department of Radiology, Maastricht University Medical Center, Maastricht, the Netherlands; 6 Department of Radiology, Gold Coast University Hospital, Gold Coast, Australia; 7 Department of Medical Imaging, Children’s Hospital of Eastern Ontario, Ottawa, ON Canada; 8 Department of Diagnostic and Interventional Radiology, “Tor Vergata” University of Rome, Rome, Italy; 9 Institute of Forensic Medicine (Virtopsy), University of Zurich, Zurich, Switzerland; 10 Los Angeles County Medical Examiner-Coroner Office, Los Angeles, CA USA; 11 Office of the Chief Medical Examiner, University of Maryland, Baltimore, MD USA; 12 Center for Cause of Death Investigation, Hokkaido University Graduate School of Medicine, Sapporo, Japan; 13 Department of Radiology, Birmingham Children’s Hospital, Birmingham, UK; 14 Department of Diagnostic Imaging & Clinical Physics, The Royal Hospital for Children, Glasgow, Scotland, UK; 15 Department of Medical Imaging, Women’s and Children’s Hospital, North Adelaide, South Australia Australia; 16 Radiology Department, National Women’s Health and Starship Children’s Hospital, Auckland City Hospital, Auckland, New Zealand; 17 Department of Forensic Medicine, Aarhus University, Aarhus, Denmark; 18 Department of Forensic Medicine, Medical University of Warsaw, Warsaw, Poland; 19 Department of Radiology, Emma Children’s Hospital, Academic Medical Center Amsterdam, Amsterdam, the Netherlands; 20 Department of Radiology and Nuclear Medicine, Radboud University Medical Center, Nijmegen, the Netherlands; 21 Academic Unit of Radiology, University of Sheffield, Sheffield, UK

**Keywords:** Children, Computed tomography, Consensus recommendations, Paediatric, Postmortem, Protocol

## Abstract

Postmortem CT for investigating childhood deaths is increasingly utilised as a noninvasive adjunct or alternative to standard autopsy; however there are no standardised published imaging protocols. This article describes a standardised imaging protocol that has been developed based on current practices of international postmortem imaging practitioners and experts. This recommendation is expected to be useful for postmortem imaging centres wishing to update their existing practices and for those starting paediatric postmortem CT as a new service.

## Introduction

Postmortem CT is a relatively fast, inexpensive and widely accessible modality, commonly used in adults as part of routine postmortem examination [1, 2]. The adaptation of this technique to evaluate childhood deaths is clearly a natural progression of the technology [3–5]. Compared to conventional paediatric autopsy practices, postmortem CT is noninvasive examination and provides a three-dimensional (3-D) digital record of anatomical information that can be post-processed in a variety of ways for clinical, research and teaching purposes. This is particularly useful where sanitised images are required for a jury in medicolegal proceedings, or to show family members when explaining the cause of death [6]. Although few publications relate to the diagnostic accuracy rates of paediatric postmortem CT, those published report reasonable concordance rates with autopsy of between 57.1% and 83.3% [4, 5, 7, 8], particularly for musculoskeletal abnormalities such as fractures.

Despite these advantages, there is much debate regarding appropriate referral indications, access to scanners, and precise techniques in performing paediatric postmortem CT. In the United Kingdom, the Royal College of Pathologists acknowledges the role that postmortem CT offers for the workup of sudden unexpected death in infancy, yet most referrals are performed on a case-by-case basis [9]. In the Netherlands, postmortem CT has been offered for all paediatric deaths by law since 2010 [10, 11]. Neither of these guidelines specifies the imaging parameters by which the examination should be conducted. A recent survey by the European Society of Paediatric Radiology (ESPR) on paediatric postmortem imaging [12] revealed that of the 24/47 (51%) centres reported to currently perform paediatric postmortem CT, only about half of these (14/24, or 58%) had a standardised CT protocol, with others reporting that protocols are modified and tailored each time for different patients. Furthermore, published studies in the literature report significant variation in image acquisition and provide insufficient information for this to be used as a template for other centres (Table 1) [3–5, 7, 8, 10, 13].

**Table 1:** Paediatric postmortem CT imaging parameters reported by published case series since 2003

Publication	Country	Computed tomography system	Coverage	Imaging parameters	Reconstruction/ algorithms	Reporter
Oyake et al. 2006 [13]	Japan	Accell Proceed, GE Medical Systems	Separate individual body parts (head or chest or abdomen)	Head: Interval: 1 cm Collimation: 1 cm Chest/Abdomen: Interval: 1 cm Collimation: 1 cm	Not mentioned	2 paediatricians + 1 radiologist
Noda et al. 2013 [5]	Japan	Asteion Super 4, Toshiba Medical Systems (4 slice)	Whole body (vertex to pelvis)	Interval: Contiguous ST: 3–8 mm	Not mentioned	2 radiologists
Proisy et al. 2013 [8]	France	Philips Brilliance (16 slice)	Whole body (vertex to feet)	120 kv, 300 mAs Collimation: 0.75 mm Pitch: 0.688 Rotation time: 0.5 s ST: 2 mm Intervals: 1 mm	Coronal, sagittal, oblique and volume-rendered reconstructions as necessary	2 paediatric radiologists
Sieswerda-Hoogendoorn et al. 2014 [7]	The Netherlands	Toshiba Aquilon (64 slice) Philips Brilliance (64 slice) Siemens Sensation (64 slice)	Whole body (limits not stated)	ST: 3 mm SFOV: Adjusted to body size	Coronal, sagittal reconstructions for all cases	1 forensic paediatric radiologist
Arthurs et al. 2016 [3]	United Kingdom	Siemens Somatom (64 slice)	Head (separate) and whole body (vertex to toes)	Head: 120 kV, variable mAs ST: 1 mm Interval: 5 mm Whole body: 120 kV, variable mAs Pitch: 1 Collimation: 0.625 mm ST: 1.25 mm	Soft-tissue and bone algorithm Volume-rendered reconstruction as needed	Paediatric radiologists (according to 5 different body part areas)
Krentz et al. 2016 [4]	Switzerland	LightSpeed8, GE Medical Systems (8 slice)	Head (separate) and whole body (vertex to feet)	Brain: 120 kV, 200–220 mAs Axial imaging mode ST: 2.5 mm Rotation: 2 s SFOV: 25 cm Body: 120 kV, 200–250 mAs Helical imaging mode ST: 0.625–1.25 mm Rotation: 0.8–1 s SFOV: 50 cm	Soft-tissue, lung and bone algorithms Multiplanar reconstructions of the spine (sagittal and coronal) Maximum-intensity projections (MIP) and volume-rendering technique as needed	Paediatric radiologist and forensic pathologist
Van Rijn et al. 2017 [10]	The Netherlands	Philips Brilliance (16- and 64-slice scanners)	Whole body acquired as separate body parts (head/neck and chest/abdomen/ extremities)	Head/neck: 120kv, 285 mAs ST: 0.9 mm Interval: 0.45 mm Pitch: 0.392 Collimation: 0.625 mm Body: 120 kV, 250 mAs ST: 3 mm Interval: 2 mm Pitch 0.983 Collimation 0.625 mm	Head/neck: Bone and brain algorithm Body: Bone and soft-tissue algorithm	One of 3 available paediatric radiologists

*SFOV* scan field of view, *ST* slice thickness

The aim of this study was therefore to develop a consensus imaging protocol for paediatric postmortem CT based on current international practices. This would enable standardisation of imaging acquisition, provide guidance for centres wishing to offer paediatric postmortem CT imaging, and aid uniformity of image quality for prospective multicentre review and research collaboration.

## Society surveys

We designed a survey containing 20 questions relating to paediatric postmortem CT imaging referral patterns, imaging acquisition parameters, reconstruction algorithms and reporting practices. Potential responses included both multiple choice answers and free-text space to allow for detailed replies and elaboration on imaging techniques. We requested that responders also submit their own local departmental paediatric postmortem CT imaging protocols for review.

All registered members of the European Society of Paediatric Radiology (ESPR) postmortem and International Society for Forensic Radiology and Imaging (ISFRI) paediatric task forces were emailed the survey in May 2016, with reminder messages sent every 3 months. We included for review all responses returned by May 2017.

## Survey findings

In total, we invited 25 imaging centres to complete the survey questionnaire. We initially received responses from 23 centres (23/25, 92%); however 3 of these centres either reported that they did not perform postmortem CT for paediatric cases, or only did so on a research basis — not as part of clinical care. Therefore, we included a total of 20 centres (20/25, 80%) in our final analysis, comprising 62 different paediatric postmortem CT protocols for review.

The surveys were completed by consultant radiologists (8/20, 40%), pathologists (6/20, 30%), both radiologists and pathologists together (2/20, 10%), radiographers/technologists (2/20, 10%) and both radiologists and radiographers together (2/20, 10%).

The responses originated from several countries, including:

- Europe (11/20, 55%) — United Kingdom (3), Denmark (1), Italy (1), the Netherlands (2), Poland (3) and Switzerland (1);
- Oceania (4/20, 20%) — Australia (3) and New Zealand (1);
- North America (4/20, 20%) — United States (3) and Canada (1); and
- Asia (1/20, 5%) — Japan (1).

The findings from the survey and pooled review of imaging protocols are described next and summarised in Tables 2 and 3.

**Table 2:** Imaging approach to paediatric postmortem CT based on survey responses from 20 centres, with consensus-recommended parameters in bold

Imaging approach	Responses *n* (%)
Scanner	
GE Healthcare	5 (25)
Philips	3 (15)
Siemens	9 (45)
Toshiba	2 (10)
Hitachi	1 (5)
Scanner location	
Hospital or clinic	9 (45)
Forensic centre/mortuary	11 (55)
Case types (not mutually exclusive)	
All paediatric cases	7 (35)
Infants (<1 year)	7 (35)
Stillbirths	2 (10)
Fetal cases	4 (20)
Other:	10 (50)
Missing information on survey	2 (10)
Special request by clinical team	3 (15)
Cause specific (e.g. hanging, abuse, trauma, burns related injuries)	5 (25)
Image interpretations	
**Radiologist**	**9 (45)**
Pathologist	3 (15)
Co-reported by radiologists and pathologists	8 (40)
Image acquisition	
**Radiographer/technologist**	**13 (65)**
Mortuary technician	3 (15)
Pathologist	3 (15)
Forensic physician (radiologist or dentist)	1 (5)
Body part imaged (not mutually exclusive)	
Whole body	12 (60)
Whole body + single anatomical area	8 (40)
Single anatomical areas (can be used to make whole-body scan)	16 (80)
Head/neck	14 (70)
Thorax, abdomen, pelvis	8 (40)
Extremities	5 (25)
Others (e.g., thorax, shoulders, pelvis–toes)	4 (20)
Energy source	
** Single-source only**	**17 (85)**
Dual-source only	1 (5)
Both single- and dual-source	2 (10)
Dose modulation	
Yes	8 (40)
**No**	**6 (30)**
On some protocols	4 (20)
Unknown	2 (10)
Detector collimator (mm)	
**0.5–1.0**	**19 (95)**
1.25	1 (5)
Image reconstructions	
Soft tissue	**19 (95)**
Bone	**20 (100)**
**Brain**	**7 (35)**
**Lung**	**9 (45)**
**Other (mediastinum, metal reduction, vendor-specific anatomical filter)**	**4 (20)**
Image reformats	
Coronal	**17 (85)**
Sagittal	**15 (75)**
Maximum-intensity projection (MIP) or minimum-intensity projection (MiniPs)	6 (30)
**Volume rendering**	**11 (55)**
Other (non-standard, special request formats)	4 (20)

**Table 3:** Paediatric postmortem CT protocol parameters based on the 62 separate CT protocols submitted, with consensus-recommended parameters in bold

Imaging parameters	Responses *n* (%)
Kilovoltage peak (kVp)	
80	3 (4.8)
100	10 (16.1)
**120**	**37 (59.7)**
130	2 (3.2)
Other (single kV not indicated)	2 (3.2)
Unknown	4 (6.4)
Dual source: 80/140–150	4 (6.4)
Milliampere seconds (mA/mAs)	
<100	7 (11.3)
100–199	6 (9.7)
**200–299**	**12 (19.4)**
300–399	8 (12.9)
400–500	9 (14.5)
Unknown	4 (6.4)
Other (range of mA/mAs listed)	2 (3.2)
Dose modulation listed (mA/mAs not defined)	11 (17.7)
Dual source 375/630 mAs	3 (4.8)
Dose modulation (within protocols)	
On	24 (38.7)
**Off**	**31 (50.0)**
Unknown	7 (11.3)
Matrix	
**512 × 512**	**26 (41.9)**
Not reported	36 (58.1)
Scan field of view (SFOV) (mm)	
≤150	1 (1.6)
>150–300	6 (9.7)
>300–450	5 (8.1)
>450 (largest reported as 750 mm)	4 (6.4)
**Adjusted to patient size**	**18 (29.0)**
Unknown	19 (30.6)
Other (extended FOV)	1 (1.6)
Scanner defined (preset based on anatomical area within the selected protocol)	8 (12.9)
Pitch	
≤0.5	3 (4.8)
**0.5–0.8**	**29 (46.8)**
0.81–0.99	13 (20.9)
1.0–1.2	4 (6.4)
>1.2 (highest reported as 1.44)	2 (3.2)
Unknown	7 (11.3)
Other (not defined, given as range 0.35–0.80)	4 (6.4)
Rotation time (s)	
<0.5	1 (1.6)
0.5	9 (14.5)
>0.5–0.99	14 (22.6)
**1.0**	**14 (22.6)**
Unknown	20 (32.3)
Other (rotation not defined, given as range 0.5–1.0; dual source 0.6, 0.5)	4 (6.4)
Slice thickness (mm)	
**≤0.75**	**34 (54.8)**
0.75–<1.0	7 (11.3)
1.0	14 (22.5)
>1.0 (largest reported as 5 mm)	3 (4.8)
Other (given as a range: 0.6–1.0 or 0.6–2.0)	3 (4.8)
Unknown	1 (1.6)

Most centres in this survey performed their imaging on a CT scanner dedicated to forensic examinations (11/20, 55%), with the remainder based in a hospital or clinic environment (9/20, 45%).

Referral indications for postmortem CT were varied, with some centres accepting all paediatric cases referred for imaging (7/20, 35%) or all infants younger than 1 year (7/20, 35%). Just less than half of all centres imaged cases referred because of a special request from the clinical team, or for a variety of specific causes (spanning a wide range of scenarios such as hanging, abuse, trauma, burn-related injuries; 5/20, 25%).

Image acquisition was mainly performed by the radiographer or technologist (13/20, 65%), although at some centres the mortuary staff (3/20, 15%), forensic physician (1/20, 5%) or pathologist (3/20, 15%) would operate the CT machinery. Image reporting was usually undertaken by a radiologist (9/20, 45%) or co-reported by a radiologist and pathologist (8/20, 40%).

There was the expected variation in the CT scanner vender and models used; however the most popular vendors included Siemens (9/20, 45%) and GE Healthcare (5/20, 25%). Almost all scanners were single-source scanners (17/20, 85%) and utilised a detector collimation of 0.5–1.0 mm (19/20, 95%).

Body coverage was variable among centres, with most performing whole-body imaging but in different ways among and within centres. Some routinely imaged the whole body in one examination (12/20, 60%, i.e. vertex to feet), whilst others also sometimes performed imaging of separate single-body regions (e.g., head, thorax, abdomen/pelvis) to make up a whole-body study, and this did not routinely include extremities (16/20, 80%). At some centres imaging of just one body region also occurred and was dependent on the referral indication (e.g., thorax for rib fractures, head/neck for traumatic brain injury).

From reviewing individual postmortem CT protocols, we noted that the following parameters were most commonly used: 120 kVp (37/62, 59.7%), 200–299 mAs (12/62, 19.4%), 0.5–0.8 pitch (29/62, 46.8%), 1-s rotation time (14/62, 22.6%) and slice thickness of ≤0.75 mm (34/62, 54.8%).

There were a variety of responses concerning scan field of view, with most protocols not specifically including this feature (19/62, 30.6%). Of those that did report the scan field of view, most protocols adjusted to patient size (attempting a scan field of view as small as possible, 18/62, 29%). Many protocols did not state matrix size (36/62, 58.1%), but where this was listed it was reported as 512 × 512 (26/62, 41.9%). The dose modulation function was stated as “off” in 50% (31/62) of cases, with the remainder of protocols either not listing this feature (7/62, 11.3%) or having this function “on” (24/62, 38.7%).

All centres reported that they routinely provided bone imaging algorithms for their postmortem CT studies, with the majority also performing soft-tissue algorithms (19/20, 95%). Many centres also provided dedicated brain (7/20, 35%) and lung (9/20, 45%) algorithms for head/neck and thoracic imaging, respectively.

In terms of multiplanar reformatting, the commonest reconstructions included coronal (17/20, 85%) and sagittal (15/20, 75%) planes. Volume-rendering reformats were provided in more than half of centres (11/20, 55%). Some centres also produced maximum- or minimum-intensity projections (6/20, 30%) or non-standard oblique reformats depending on pathology and special request (4/20, 20%).

## Consensus formation

Survey responses were presented at the postmortem imaging task force session of the annual ESPR conference in June 2017 in Davos, Switzerland. Based on the survey results, a recommended paediatric postmortem CT imaging protocol was proposed at this meeting, developed by the leading authors. The final version of this manuscript was circulated among members of ISFRI and ESPR in September 2018 for consensus approval and was formally endorsed by both the ISFRI and ESPR board prior to submission for publication.

## Recommended protocol

Based on described current practices using the most commonly reported imaging parameters, a paediatric postmortem CT protocol has been devised and is shown in Table 4. This could be easily achieved with minor modifications to most CT scanner models from all vendors at most centres.

**Table 4:** Recommended paediatric postmortem CT imaging protocol

Reporting and referrals	
Case types	Practitioner-dependent and case-specific
Image acquisition	Performed by trained radiographer
Image interpretation	Performed by qualified radiologist
Image acquisition	
Coverage	Whole body (vertex to extremities)
Kilovoltage peak (kVp)	120
Milliampere seconds (mAs)	200–299
Pitch	0.5–0.8
Slice thickness (mm)	≤0.75
Energy source	Single source
Dose modulation	Off
Scan field of view (SFOV) (mm)	Adjust to patient size (small as possible)
Detector collimator (mm)	0.5–1.0
Rotation time (s)	1.0
Matrix	512 × 512
Kernel/filter/algorithm	Soft-tissue and bone (whole body) Brain (brain coverage) Lung (thoracic coverage)
Reformats	Coronal, sagittal, volume rendering

**Fig. 1:**
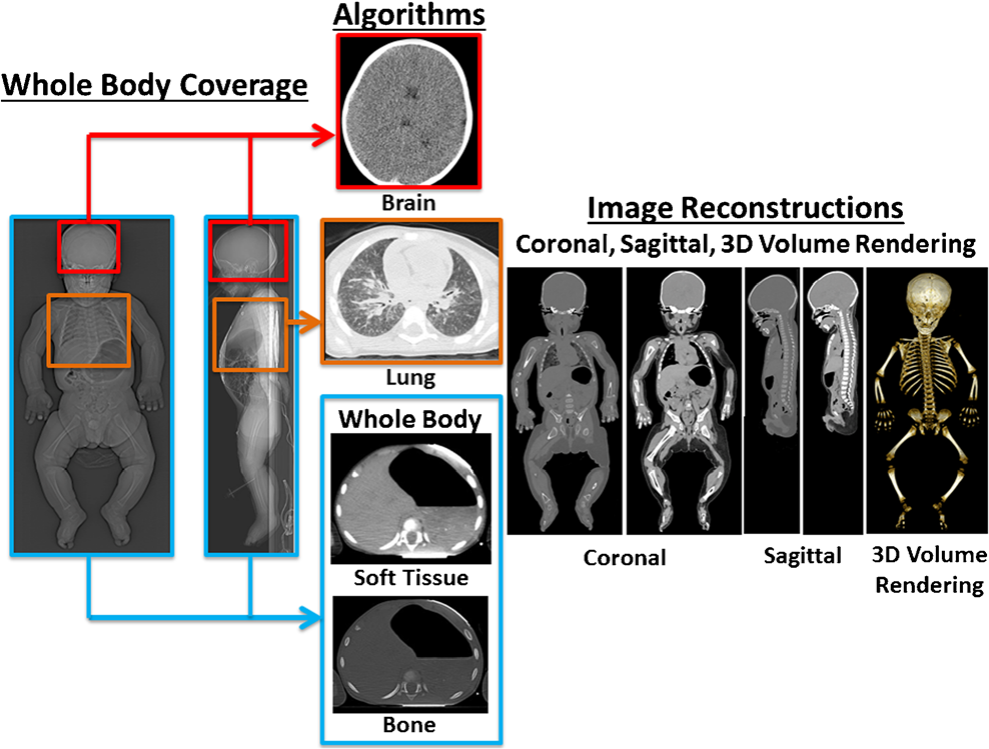
Representation of the recommended joint European Society of Paediatric Radiology (ESPR) and International Society for Forensic Radiology and Imaging (ISFRI) postmortem CT imaging body coverage. The blue box represents the whole-body coverage and area for application of soft-tissue and bone algorithms. The red box represents the region for application of the brain algorithm, and the purple box the lung algorithm. Image reconstruction planes (both coronal and sagittal) are included

We provide a pictorial representation of recommended whole-body scan coverage, which could be printed and posted within the CT control room as a reminder of how to perform a paediatric postmortem CT (Fig. 1). For ease of scanning we recommend a single scan through the whole body using the recommended imaging parameters (Table 4), with separate reconstruction algorithms for brain and body.

Whilst imaging of the extremities during paediatric postmortem CT is achievable and recommended, this is predominantly for bone assessment in the setting of trauma and might not be necessary where good-quality skeletal radiographs are available.

## Discussion

The survey revealed common practices of paediatric postmortem CT imaging at several international centres with significant experience in forensic and postmortem imaging. Our paediatric postmortem CT protocol has been recommended and agreed in consensus, which allows for future standardisation of image acquisition, improved ease of reporting — particularly when requesting external reviewer or expert opinion on cases — as well as multicentre collaborations.

Compared to several large paediatric case series utilising postmortem CT imaging dating to 2006 (Table 1), our recommended imaging parameters fall readily within those being performed worldwide. The kilovoltage of 120 kV, current of 200–299 mAs, detector collimation of 0.5–1.0 cm and rotation time of 1 s are already in common practice. It is also common practice for CT imaging to be reported by trained radiologists, and for the appropriate bone and soft-tissue algorithms to be used. Many published studies also reported routinely reviewing multiplanar reconstructions (i.e. coronal, sagittal planes) as well as volume-rendering reformats.

The main differences between our recommendations and the published literature include methods by which whole-body coverage is achieved, and image slice thickness. For body coverage, some papers report imaging from vertex to feet [8], others from vertex to pelvis [5]; some image the head and body separately [3, 4, 10] or individual body areas, as required [13]. For simplicity and ease of adopting this protocol, we recommend imaging the entire body (from vertex to extremities) in a single examination, rather than imaging the head and neck in one examination and the thorax, abdomen, pelvis and lower limbs as a second separate examination. It should be noted, however, that whilst imaging the extremities is useful in situations relating to major trauma or obvious external injuries, the reduced inherent resolution of the CT examination compared to plain radiography can result in missing subtle fractures such as classic metaphyseal lesions. Therefore, if there is concern for physical abuse, one should not dismiss performing additional radiographs (particularly of the limbs). To the best of our knowledge, no studies have assessed diagnostic accuracy rates of CT imaging versus plain radiography in the identification of classic metaphyseal lesions, although from anecdotal experience this is well recognised and might represent an area for further evidence-based research.

We recommend a slice thickness of ≤0.75 mm, whereas several reported studies in the literature used slice thicknesses ranging 1–10 mm. This might have been a result of limitations of older scanner models, which are likely to have now been updated within most hospitals and forensic centres. Nevertheless, where centres exist with models that cannot achieve slice thicknesses below 0.75 mm, we suggest imaging at the thinnest possible slice on the equipment available.

One factor we include in our recommended protocol that is not specifically mentioned within the published literature relates to non-usage of the dose modulation function (otherwise known as automated tube current modulation or automatic exposure control). The primary advantage of this technique is the ability to reduce the radiation dosage from a CT study without significantly influencing image quality. This is achieved by automatically adjusting the tube current with differences in soft-tissue attenuation and the body part being scanned [14]. Whilst this offers obvious advantages when imaging live children [15], these risks do not apply in the postmortem population. Adjusting and varying the tube current unnecessarily, especially using different methods of dose modulation by different CT vendors, might therefore introduce variability in signal to noise ratio and thus image quality [16]. In our review of paediatric postmortem CT protocols, this is already “off” in 50% of cases and should not be used for paediatric postmortem CT imaging.

As with all studies, our retrospective self-reported study has several inherent limitations. The first relates to the relatively small number, and apparent European bias in the centres surveyed. Despite this, our results reflect the best expertise available given the high response rate from task force members from two international societies specifically established to examine this issue. Further, most published studies on paediatric postmortem CT imaging stem from Europe (Table 1).

The second limitation relates to the survey questions. These questions were designed to be concise, easy to understand and relevant to postmortem CT imaging parameters. We acknowledge that there are many other aspects surrounding the running of a paediatric postmortem CT imaging service that would have been interesting and useful to have studied. These could have included further details on imaging referral patterns and case numbers, reporting environment (e.g., standardised reporting, double reporting, presence of multi-disciplinary team meetings) and issues regarding postmortem imaging training and reporting (e.g., years of experience of technologists and reporters). Although this would have been valuable to collect, we believe they would have made the survey more time-consuming to complete and threaten to lower our response rates from society members. Furthermore, these questions would have detracted from the main purpose of the survey, which was to produce recommendations specifically for postmortem CT imaging parameters. Many of the other issues described therefore fall outside this remit.

Finally, our recommendations are based on the current common practices of the majority of centres. To our knowledge no published CT imaging optimisation studies show a direct comparison between image quality or diagnostic accuracy with different parameters in paediatric postmortem cases. Our recommendations are therefore based on a pragmatic approach, easily adapted for a range of patient sizes and ages and easily implemented on most if not all CT scanners. The recommendations are intended to standardise paediatric postmortem CT imaging acquisition to allow for consistent image quality for diagnosis and multicentre sharing of data for future research collaboration studies. It also offers centres wishing to start paediatric postmortem CT as a new service a guide for setting up their local protocols.
